# Supplementary material for: Influenza Vaccine Uptake in Italy—The 2022–2023 Seasonal Influenza Vaccination Campaign in Italy: An Update from the OBVIOUS Project
Source: Vaccines (Basel). 2024 Mar 12;12(3):297. doi: 10.3390/vaccines12030297 (PMC10974551; doi:10.3390/vaccines12030297)
Supplement: Supplementary file 1 [file vaccines-12-00297-s001.zip › Supplemental Material S1.docx.pdf]

1. What is your gender?
   - Male
   - Female
   - Non-Binary
   - Prefer not to answer

2. Please indicate your date of birth:
   - DD/MM/YYYY

(Note: Respondents under 18 years old are screened out.)

3. In which region do you live?
   - [List of Italian regions from Abruzzo to Veneto]
4. In which province?
5. In which municipality?
6. Please indicate your level of education:
   - Primary/Middle school certificate
   - High school diploma (high school, art/technical/professional school, teacher training school)
   - Degree (pre-reform, first or second level, single cycle) or academic diploma
   - Postgraduate qualification (advanced training course, university master, specialization school, research doctorate)
7. What is your current occupation?
   - Student
   - Doctor
   - Other healthcare worker
   - Law enforcement
   - Teacher
   - Unemployed
   - Retired
   - Employed (other category)
8. Who do you live with?
   - Alone
   - With a partner
   - With my family of origin
   - Other
9. With the financial resources at your disposal (from your own or family income), can you meet the needs of your current life situation?

   Answer scale (randomized order):

   - Very easily
   - Fairly easily
   - With some difficulty
   - With great difficulty
10. Do you have children?
    - Yes
    - No

    If Yes: 11) What is the gender of your youngest child? - Male - Female

12. Please indicate the date of birth of your youngest child:
    - DD/MM/YYYY
13. Who makes the decisions about vaccinating your children?
    - Mostly me
    - Mostly my partner
    - Equally divided

11. Were you pregnant at the beginning of the flu season (October/November 2022)?
    - Yes
    - No
12. Due to a physical/psychological/sensory disability, do you have difficulty completing daily tasks such as going to the doctor or shopping?
    - Yes
    - No
13. Indicate your weight:
    - _ _ _ kg
14. Indicate your height in centimeters:
    - _ _ _ cm
15. Please indicate if you suffer from any of the following diseases:
    - Chronic respiratory diseases (including severe asthma, bronchopulmonary dysplasia, cystic fibrosis, and chronic obstructive pulmonary disease)
    - Chronic diseases of the cardiovascular system (including congenital and acquired heart diseases)
    - Diabetes

    Answers:

    - Yes
    - No
16. Where have you had most of your vaccinations done?
    - In a hospital
    - In a pharmacy
    - By a family doctor
    - In a vaccination hub
    - At home
    - At the workplace
17. If you could choose, where would you prefer to receive a vaccination?
    - [Same options as question 20]

21a) At what time of day, according to your commitments, would you prefer to receive any vaccinations?

- From 6 to 9 in the morning

- From 9 to 12 in the morning

- From 12 to 3 in the afternoon

- From 3 to 6 in the afternoon

- From 6 to 9 in the evening

21b) On which day of the week, according to your commitments, would you prefer to receive any vaccinations?

- [Days of the week from Monday to Sunday]

22. What do your family and friends think of vaccinations?

    Answer scale (randomized order):

    - Very unfavorable
    - Unfavorable
    - Fairly unfavorable
    - Fairly favorable
    - Favorable
    - Very favorable

22b) Where/who do you get information from to know which vaccinations can be directed at you?

- Family doctor/pediatrician

- Newspapers

- Internet

- TV

- Friends/relatives

- Other health professionals

INFLUENZA SPECIFIC SECTION

[Square Brackets show modified, additional, questions addressed to parents who reported to have at least a child].

23. Did you[r child] get the flu vaccine during the October-December 2022 season?

- Yes
- No
- Not sure (If 'Not sure', skip to the next relevant section)

24. Do you intend to get the flu vaccine? [Are you planning to have your youngest child vaccinated against the flu?]

- Yes
- No

25. How concerned are you about [your youngest child] getting the flu?

Answer scale (randomized order):

- Not at all concerned
- Slightly concerned
- Moderately concerned
- Very concerned

26. How safe do you think the flu vaccine is [for your youngest child]?

Answer scale (randomized order):

- Not at all safe
- Slightly safe
- Moderately safe
- Very safe

27. Do you think you[r youngest child] are [is] eligible to receive the flu vaccine?

- Yes
- No
- Don't know

28a) If a relative/friend were offered the flu vaccine, what would you advise them to do?

- Get it
- Don't get it
- Don't know

28b) Who suggested getting the flu vaccine [for your youngest child]?

- Local Health Authority via letter/call/email
- Family doctor/ Pediatrician
- At the workplace (by the occupational doctor)
- Gynecologist (if pregnant)
- Obstetrician (if pregnant)
- Other healthcare professionals
- No one

28c) Did you found it difficult accessing the flu vaccine [for your youngest child]?

- Yes, I did
- No, I did not

28d) If you wanted to get the flu vaccine [for your youngest child], would you know how to obtain it?

- Yes, I would know how to get it
- No, I wouldn't know how to get it

28e) If you were pregnant and had the flu vaccine, what week of pregnancy were you in when you had it?

- [Dropdown menu from week 1 to 42]
- I had it before I knew I was pregnant
