# Supplementary material for: Influenza Vaccine Uptake in Italy—The 2022–2023 Seasonal Influenza Vaccination Campaign in Italy: An Update from the OBVIOUS Project
Source: Vaccines (Basel). 2024 Mar 12;12(3):297. doi: 10.3390/vaccines12030297 (PMC10974551; doi:10.3390/vaccines12030297)
Supplement: Supplementary file 1 [file vaccines-12-00297-s001.zip › SUPPLEMENTALS tables & figures.docx.pdf]

Supplementary materials

Supplemental Table S-1. Sociodemographic characteristics of the study respondents who provided information about their youngest children's seasonal influenza vaccine uptake, overall and by NUTS statistical region.

Characteristic	Italy ($n = 437$)	Northwestern Italy ($n = 93$)	Northeastern Italy ($n = 75$)	Central Italy ($n = 86$)	Southern Italy ($n = 118$)	Insular Italy ($n = 65$)
Gender						
Male	200 (45.8%)	42 (45.2%)	38 (50.7%)	37 (43.0%)	59 (50.0%)	24 (36.9%)
Female	237 (54.2%)	51 (54.8%)	37 (49.3%)	49 (57.0%)	59 (50.0%)	41 (63.1%)
Age, y						
Mean ± SD	36.9 ± 6.6	36.7 ± 7.8	37.5 ± 7.0	36.9 ± 5.6	37.1 ± 5.8	36.2 ± 6.7
Median [IQR]	37 [33–42]	37 [32–42]	37 [33–43]	37.5 [34–40]	37 [33–42]	36 [32–42]
Age group, y						
18–24	13 (3.0%)	4 (4.3%)	1 (1.3%)	2 (2.3%)	3 (2.5%)	3 (4.6%)
25–34	124 (28.4%)	29 (31.2%)	21 (28.0%)	20 (23.3%)	32 (27.1%)	22 (33.8%)
35–44	242 (55.4%)	44 (47.3%)	38 (50.7%)	56 (65.1%)	73 (61.9%)	31 (47.7%)
45–54	55 (12.6%)	15 (16.1%)	14 (18.7%)	8 (9.3%)	9 (7.6%)	9 (13.8%)
55–64	3 (0.7%)	1 (1.1%)	1 (1.3%)	0 (0.0%)	1 (0.8%)	0 (0.0%)
Place of residence degree of urbanization						
City (densely populated area)	136 (31.1%)	35 (37.6%)	10 (13.3%)	30 (34.9%)	50 (42.4%)	11 (16.9%)
Town or suburb (intermediate density area)	213 (48.7%)	42 (45.2%)	43 (57.3%)	40 (46.5%)	46 (39.0%)	42 (64.6%)
Rural area (thinly populated area)	88 (20.1%)	16 (17.2%)	22 (29.3%)	16 (18.6%)	22 (18.6%)	12 (18.5%)
Educational attainment						
Less than high school diploma	40 (9.2%)	11 (11.8%)	6 (8.0%)	4 (4.7%)	11 (9.3%)	8 (12.3%)
High school diploma	261 (59.7%)	55 (59.1%)	52 (69.3%)	50 (58.1%)	63 (53.4%)	41 (63.1%)
Academic degree	102 (23.3%)	20 (21.5%)	12 (16.0%)	26 (30.2%)	30 (25.4%)	14 (21.5%)
Post-graduate/Doctorate degree	34 (7.8%)	7 (7.5%)	5 (6.7%)	6 (7.0%)	14 (11.9%)	2 (3.1%)
Household composition						
Alone	10 (2.3%)	2 (2.2%)	2 (2.7%)	2 (2.3%)	3 (2.5%)	1 (1.5%)
Couple	348 (79.6%)	75 (80.6%)	56 (74.7%)	68 (79.1%)	98 (83.1%)	51 (78.5%)
Family of origin	36 (8.2%)	9 (9.7%)	9 (12.0%)	8 (9.3%)	5 (4.2%)	5 (7.7%)
Other	43 (9.8%)	7 (7.5%)	8 (10.7%)	8 (9.3%)	12 (10.2%)	8 (12.3%)
Able to pay for things needed in life						
With great difficulty	49 (11.2%)	7 (7.5%)	6 (8.0%)	10 (11.6%)	15 (12.7%)	11 (16.9%)

With some difficulty	221 (50.6%)	44 (47.3%)	40 (53.3%)	44 (51.2%)	60 (50.8%)	33 (50.8%)
Quite easily	148 (33.9%)	38 (40.9%)	24 (32.0%)	30 (34.9%)	37 (31.4%)	19 (29.2%)
Easily	19 (4.3%)	4 (4.3%)	5 (6.7%)	2 (2.3%)	6 (5.1%)	2 (3.1%)

Notes: Northwestern Italy includes the regions of Piedmont, Aosta Valley, Lombardy, and Liguria; Northeastern Italy includes the regions of Trentino-South Tyrol, Veneto, Friuli-Venezia Giulia, and Emilia-Romagna; Central Italy includes the regions of Tuscany, Umbria, Marche, and Lazio; Southern Italy includes the regions of Abruzzo, Molise, Campania, Apulia, Basilicata, and Calabria; Insular Italy includes the regions of Sicily and Sardinia.
Abbreviations: NUTS, Nomenclature of Territorial Units for Statistics.

Supplemental Table S-2. Clinical characteristics of the study respondents who provided information about their own seasonal influenza vaccine uptake, overall and by NUTS statistical region.

Characteristic	Italy (*n* = 5217)	Northwestern Italy (*n* = 1517)	Northeastern Italy (*n* = 998)	Central Italy (*n* = 1011)	Southern Italy (*n* = 1151)	Insular Italy (*n* = 540)
Pregnant in October/November 2022						
Yes	284 (5.4%)	76 (5.0%)	52 (5.2%)	56 (5.5%)	68 (5.9%)	32 (5.9%)
No	2376 (45.5%)	751 (49.5%)	374 (37.5%)	490 (48.5%)	495 (43.0%)	266 (49.3%)
Not applicable	2557 (49.0%)	690 (45.5%)	572 (57.3%)	465 (46.0%)	588 (51.1%)	242 (44.8%)
Problems with daily living tasks due to physical or mental impairment						
Yes	688 (13.2%)	139 (9.2%)	204 (20.4%)	127 (12.6%)	153 (13.3%)	65 (12.0%)
No	4529 (86.8%)	1378 (90.8%)	794 (79.6%)	884 (87.4%)	998 (86.7%)	475 (88.0%)
BMI $\geq$ 30 kg/m²						
Yes	1171 (22.4%)	285 (18.8%)	203 (20.3%)	258 (25.5%)	282 (24.5%)	143 (26.5%)
No	4046 (77.6%)	1232 (81.2%)	795 (79.7%)	753 (74.5%)	869 (75.5%)	397 (73.5%)
Pneumopathy						
Yes	537 (10.3%)	125 (8.2%)	81 (8.1%)	111 (11.0%)	151 (13.1%)	69 (12.8%)
No	4680 (89.7%)	1392 (91.8%)	917 (91.9%)	900 (89.0%)	1000 (86.9%)	471 (87.2%)
Cardiopathy						
Yes	638 (12.2%)	184 (12.1%)	97 (9.7%)	121 (12.0%)	165 (14.3%)	71 (13.1%)
No	4579 (87.8%)	1333 (87.9%)	901 (90.3%)	890 (88.0%)	986 (85.7%)	469 (86.9%)
Diabetes						
Yes	951 (18.2%)	247 (16.3%)	260 (26.1%)	150 (14.8%)	205 (17.8%)	89 (16.5%)
No	4266 (81.8%)	1270 (83.7%)	738 (73.9%)	861 (85.2%)	946 (82.2%)	451 (83.5%)

Notes: Northwestern Italy includes the regions of Piedmont, Aosta Valley, Lombardy, and Liguria; Northeastern Italy includes the regions of Trentino-South Tyrol, Veneto, Friuli-Venezia Giulia, and Emilia-Romagna; Central Italy includes the regions of Tuscany, Umbria, Marche, and Lazio; Southern Italy includes the regions of Abruzzo, Molise, Campania, Apulia, Basilicata, and Calabria; Insular Italy includes the regions of Sicily and Sardinia.
Abbreviations: NUTS, Nomenclature of Territorial Units for Statistics; BMI, body mass index.

Supplemental Table S-3. Perception of the safety of seasonal influenza vaccines among respondents who answered on their own behalf, overall and by gender and NUTS statistical region.

	All	Very safe	Quite safe	Quite unsafe	Very unsafe
Males and females					
Italy	5217 (100.0%)	1207 (23.1%)	2916 (55.9%)	798 (15.3%)	296 (5.7%)
Northwestern Italy	1517 (100.0%)	378 (24.9%)	825 (54.4%)	221 (14.6%)	93 (6.1%)
Northeastern Italy	998 (100.0%)	253 (25.4%)	562 (56.3%)	134 (13.4%)	49 (4.9%)
Central Italy	1011 (100.0%)	223 (22.1%)	579 (57.3%)	157 (15.5%)	52 (5.1%)
Southern Italy	1151 (100.0%)	244 (21.2%)	654 (56.8%)	189 (16.4%)	64 (5.6%)
Insular Italy	540 (100.0%)	109 (20.2%)	296 (54.8%)	97 (18.0%)	38 (7.0%)
Males					
Italy	2557 (100.0%)	661 (25.9%)	1414 (55.3%)	325 (12.7%)	157 (6.1%)
Northwestern Italy	690 (100.0%)	196 (28.4%)	379 (54.9%)	77 (11.2%)	38 (5.5%)
Northeastern Italy	572 (100.0%)	157 (27.4%)	319 (55.8%)	67 (11.7%)	29 (5.1%)
Central Italy	465 (100.0%)	112 (24.1%)	262 (56.3%)	58 (12.5%)	33 (7.1%)
Southern Italy	588 (100.0%)	143 (24.3%)	329 (56.0%)	81 (13.8%)	35 (6.0%)
Insular Italy	242 (100.0%)	53 (21.9%)	125 (51.7%)	42 (17.4%)	22 (9.1%)
Females					
Italy	2660 (100.0%)	546 (20.5%)	1502 (56.5%)	473 (17.8%)	139 (5.2%)
Northwestern Italy	827 (100.0%)	182 (22.0%)	446 (53.9%)	144 (17.4%)	55 (6.7%)
Northeastern Italy	426 (100.0%)	96 (22.5%)	243 (57.0%)	67 (15.7%)	20 (4.7%)
Central Italy	546 (100.0%)	111 (20.3%)	317 (58.1%)	99 (18.1%)	19 (3.5%)
Southern Italy	563 (100.0%)	101 (17.9%)	325 (57.7%)	108 (19.2%)	29 (5.2%)
Insular Italy	298 (100.0%)	56 (18.8%)	171 (57.4%)	55 (18.5%)	16 (5.4%)

Notes: Females include non-binary persons. Northwestern Italy includes the regions of Piedmont, Aosta Valley, Lombardy, and Liguria; Northeastern Italy includes the regions of Trentino-South Tyrol, Veneto, Friuli-Venezia Giulia, and Emilia-Romagna; Central Italy includes the regions of Tuscany, Umbria, Marche, and Lazio; Southern Italy includes the regions of Abruzzo, Molise, Campania, Apulia, Basilicata, and Calabria; Insular Italy includes the regions of Sicily and Sardinia.

Abbreviations: NUTS, Nomenclature of Territorial Units for Statistics.

Supplemental Table S-4. Perception of the safety of seasonal influenza vaccines by high-risk target group based on age, clinical status or profession.

	All	Very safe	Quite safe	Quite unsafe	Very unsafe
Ages ≥ 60 y	2839 (100.0%)	706 (24.9%)	1633 (57.5%)	367 (12.9%)	133 (4.7%)
Children	437 (100.0%)	82 (18.8%)	228 (52.2%)	96 (22.0%)	31 (7.1%)
Pregnant women	284 (100.0%)	53 (18.7%)	162 (57.0%)	53 (18.7%)	16 (5.6%)
People with diabetes	951 (100.0%)	264 (27.8%)	488 (51.3%)	134 (14.1%)	65 (6.8%)
People with CVDs	638 (100.0%)	157 (24.6%)	341 (53.4%)	104 (16.3%)	36 (5.6%)
People with RDs	537 (100.0%)	123 (22.9%)	290 (54.0%)	85 (15.8%)	39 (7.3%)
People with BMI ≥ 30 kg/m²	1171 (100.0%)	233 (19.9%)	653 (55.8%)	213 (18.2%)	72 (6.1%)
Medical doctors	92 (100.0%)	32 (34.8%)	41 (44.6%)	16 (17.4%)	3 (3.3%)
Other HC workers	279 (100.0%)	69 (24.7%)	144 (51.6%)	49 (17.6%)	17 (6.1%)
Teachers	434 (100.0%)	93 (21.4%)	257 (59.2%)	62 (14.3%)	22 (5.1%)
Law enforcement members	136 (100.0%)	24 (17.6%)	67 (49.3%)	34 (25.0%)	11 (8.1%)

Notes: Information about children was provided by their parents.
Abbreviations: CVD, cardiovascular disease; RD, respiratory disease; BMI, body mass index; HC, healthcare.

Supplemental Table S-5. Results of multivariable multinomial logistic regression analysis: determinants of seasonal influenza vaccine uptake and hesitancy (expressed as delay vs. refusal) among respondents who answered on their children's behalf (*n* = 437).

Characteristic	Did get the vaccine			Would get the vaccine			Would not get the vaccine		
	Predicted probability	Discrete difference (*Δ*)		Predicted probability	Discrete difference (*Δ*)		Predicted probability	Discrete difference (*Δ*)	
		Estimate	95% CI		Estimate	95% CI		Estimate	95% CI
Parent's gender									
Male	31.3%	Ref.		11.6%	Ref.		57.1%	Ref.	
Female	24.8%	−6.4	−13.0, 0.1	15.8%	4.3	−2.1, 10.6	59.3%	2.2	−4.7, 9.1
Parent's age group, y									
18–34	28.3%	Ref.		15.7%	Ref.		56.0%	Ref.	
35–44	25.6%	−2.6	−9.7, 4.4	13.9%	−1.8	−8.8, 5.1	60.5%	4.5	−3.1, 12.0
45–59	37.4%	9.1	−1.5, 19.8	8.6%	−7.1	−16.6, 2.4	53.9%	−2.0	−12.7, 8.7

NUTS statistical region									
Northwestern Italy	32.4%	Ref.		12.6%	Ref.		55.1%	Ref.	
Northeastern Italy	19.9%	−12.4*	−23.4, −1.4	20.2%	7.6	−4.4, 19.7	59.9%	4.8	−6.8, 16.5
Central Italy	28.7%	−3.7	−13.2, 5.9	8.5%	−4.0	−12.9, 4.8	62.8%	7.7	−2.4, 17.8
Southern Italy	28.6%	−3.7	−13.1, 5.6	16.0%	3.4	−5.9, 12.7	55.4%	0.3	−9.5, 10.1
Insular Italy	25.3%	−7.0	−18.3, 4.2	15.2%	2.6	−8.3, 13.6	59.5%	4.4	−7.4, 16.2
Degree of urbanization									
City	26.6%	Ref.		17.3%	Ref.		56.2%	Ref.	
Town or suburb	27.7%	1.1	−6.3, 8.5	12.7%	−4.5	−12.2, 3.2	59.6%	3.4	−4.6, 11.4
Rural area	31.4%	4.8	−4.7, 14.4	11.9%	−5.4	−14.5, 3.7	56.7%	0.6	−9.4, 10.5
Parent’s educational attainment									
Academic/Post-graduate degree	30.9%	Ref.		10.5%	Ref.		58.6%	Ref.	
Up to high school diploma	26.2%	−4.7	−11.9, 2.4	15.8%	5.3	−1.2, 11.8	58.0%	−0.6	−7.8, 6.6
Child’s gender									
Male	27.9%	Ref.		12.7%	Ref.		59.3%	Ref.	
Female	27.8%	−0.2	−6.8, 6.4	15.5%	2.8	−3.5, 9.1	56.7%	−2.6	−9.3, 4.1
Worry about seasonal influenza									
Very/Quite worried	33.8%	Ref.		22.2%	Ref.		44.0%	Ref.	
A little worried	23.5%	−10.4*	−17.8, −2.9	11.0%	−11.2*	−19.3, −3.1	65.6%	21.6*	12.8, 30.3
Not worried	29.7%	−4.1	−16.2, 8.0	4.2%	−17.9*	−27.0, −8.9	66.0%	22.0*	9.5, 34.6
Perception of vaccine safety for the child									
Very safe	36.0%	Ref.		10.7%	Ref.		53.3%	Ref.	
Quite safe	30.0%	−6.0	−15.5, 3.5	15.7%	5.1	−2.3, 12.4	54.3%	0.9	−8.9, 10.8
Quite/Very unsafe	12.6%	−23.4*	−35.8, −11.1	17.4%	6.7	−4.8, 18.2	70.1%	16.7*	2.9, 30.5
Dear ones’ views on vaccination in general									
Very favorable	25.5%	Ref.		16.5%	Ref.		58.0%	Ref.	
Favorable	26.8%	1.3	−7.9, 10.5	15.0%	−1.5	−11.1, 8.1	58.2%	0.2	−10.0, 10.4
Quite favorable	30.2%	4.7	−4.3, 13.6	10.8%	−5.6	−14.7, 3.4	58.9%	1.0	−9.2, 11.2
Quite to very unfavorable	28.5%	2.9	−9.7, 15.5	16.8%	0.3	−13.6, 14.2	54.7%	−3.2	−17.1, 10.6
Awareness that the child has priority for vaccination									
Yes	31.8%	Ref.		11.9%	Ref.		56.3%	Ref.	
No	16.1%	−15.7*	−25.8, −5.6	18.4%	6.4	−4.3, 17.2	65.6%	9.3	−1.7, 20.2
Don’t know	21.1%	−10.7*	−19.4, −2.0	19.0%	7.1	−1.2, 15.4	59.9%	3.6	−4.7, 11.9
Advice to friends/relatives invited for vaccination									
Get it	30.9%	Ref.		20.1%	Ref.		49.0%	Ref.	
Don’t get it	34.3%	3.4	−8.7, 15.6	1.5%	−18.7*	−24.9, −12.4	64.2%	15.2*	2.6, 27.8
Don’t know	14.2%	−16.7*	−25.7, −7.7	9.0%	−11.1*	−19.9, −2.3	76.8%	27.8*	17.4, 38.3

Who invited for vaccination									
No one	5.4%	Ref.		13.6%	Ref.		80.9%	Ref.	
Local Healthcare Authority	34.6%	29.2*	15.1, 43.3	10.0%	−3.7	−15.0, 7.7	55.4%	−25.5*	−40.9, −10.2
Pediatrician	33.5%	28.1*	20.1, 36.0	18.2%	4.5	−4.3, 13.4	48.3%	−32.6*	−42.6, −22.6
Other medical doctors	61.7%	56.3*	39.5, 73.0	14.7%	1.1	−12.2, 14.3	23.6%	−57.3*	−72.0, −42.6

*P-value ≤0.05, that is, Δ significantly ≠ 0.

Abbreviations: NUTS, Nomenclature of Territorial Units for Statistics.
